# Discovery of cerebrospinal fluid biomarkers for different dementias using mass spectrometry‐based proteomics

**DOI:** 10.1002/dad2.70278

**Published:** 2026-04-12

**Authors:** Marijke E. Stokkel, Lisa Vermunt, Jaco C. Knol, Davide Chiasserini, Lucilla Parnetti, Sander R. Piersma, Thang V. Pham, Richard R. de Goeij‐de Haas, Afina W. Lemstra, Yolande A. L. Pijnenburg, Pieter J. Visser, Betty M. Tijms, Charlotte E. Teunissen, Connie R. Jimenez

**Affiliations:** ^1^ Alzheimer Center Amsterdam Department of Neurology Vrije Universiteit Amsterdam, Amsterdam UMC location VUmc Amsterdam Noord‐Holland the Netherlands; ^2^ Amsterdam Neuroscience Neurodegeneration Amsterdam Noord‐Holland the Netherlands; ^3^ Department of Laboratory Medicine Neurochemistry Laboratory Vrije Universiteit Amsterdam, Amsterdam UMC location VUmc Amsterdam Noord‐Holland the Netherlands; ^4^ Department of Medical Oncology Vrije Universiteit Amsterdam Amsterdam UMC location VUmc Amsterdam Noord‐Holland the Netherlands; ^5^ OncoProteomics Laboratory Amsterdam UMC location VUmc Amsterdam Noord‐Holland the Netherlands; ^6^ Department of Medicine and Surgery Section of Physiology and Biochemistry University of Perugia Perugia Italy; ^7^ Department of Medicine and Surgery Section of Neurology University of Perugia Perugia Italy; ^8^ Department of Psychiatry Maastricht University Maastricht Limburg the Netherlands

**Keywords:** Alzheimer's disease, cerebrospinal fluid, dementia with Lewy bodies, fluid biomarker, frontotemporal dementia, mass spectrometry, mild cognitive impairment, proteomics

## Abstract

**INTRODUCTION:**

Common forms of dementia include Alzheimer's disease (AD), dementia with Lewy bodies (DLB), and frontotemporal dementia (FTD). Disease specific biomarkers are needed for differential disease diagnosis.

**METHODS:**

We performed cerebrospinal fluid (CSF) mass spectrometry proteomics on three cohorts (*n* = 110, *n* = 112, *n* = 78) including AD, DLB, FTD, mild cognitive impairment (MCI) with or without abnormal cerebrospinal fluid (CSF) amyloid‐beta 1–42 (Aβ_1–42_) levels (MCI Aβ+ and MCI Aβ‐, respectively) and controls.

**RESULTS:**

We identified and validated 11, 3, and 5 differentially expressed proteins in AD, DLB, and FTD, respectively. Potential disease specific proteins included fructose‐bisphosphate aldolase A (ALDOA), L‐lactate dehydrogenase A chain (LDHA), malate dehydrogenase, cytoplasmic (MDH1), and phosphoglycerate mutase 1 (PGAM1), which were upregulated in AD and MCI Aβ+ across multiple cohorts and did not display altered levels in DLB and FTD. Validated DLB and FTD proteins were altered in similar directions in other dementia types in at least one other cohort.

**DISCUSSION:**

Proteomics identified potential disease specific biomarkers in AD which were already altered in the prodromal stage.

**Highlights:**

- We studied cerebrospinal fluid (CSF) proteomic alterations in Alzheimer's disease (AD), dementia with Lewy bodies (DLB), and frontotemporal dementia (FTD).
- Proteins with altered CSF levels were associated with immune related processes in AD, DLB, and FTD, glycolytic processes in AD and amyloid‐beta positive mild cognitive impairment (MCI Aβ+), and synaptic processes in FTD.
- MCI Aβ+ and MCI Aβ‐ displayed divergent proteomic changes, with MCI Aβ+ being more similar to AD, while alterations in MCI Aβ‐ were difficult to relate to any of the dementias studied.
- Glycolytic protein levels were specifically upregulated in AD and MCI Aβ+ in our cohorts and in previously published cohorts, while these were unaltered in DLB and FTD.

## 1 BACKGROUND

Dementia is characterized by cognitive decline and presents a high burden on aging populations. Common forms of dementia include Alzheimer's disease (AD), dementia with Lewy bodies (DLB), and frontotemporal dementia (FTD). Pathologically, these dementias are defined by aggregation of amyloid‐beta (Aβ) and tau in AD, α‐synuclein in DLB, and TAR DNA‐binding protein 43 (TDP‐43) and/or tau in FTD. Overlap in clinical symptoms severely impacts the accuracy with which these dementias can be diagnosed,^1^, ^2^ underscoring the need for disease specific biomarker development.

Proteomic profiling of cerebrospinal fluid (CSF) is a powerful approach to study the pathophysiology of neurodegenerative disease and identify potential novel biomarkers. Data independent acquisition mass spectrometry (DIA‐MS) is particularly useful, as it allows for untargeted proteomic profiling with fewer missing values and higher reproducibility compared to other MS methods.^3^, ^4^ Studies utilizing this technique have identified biological process enrichment of glycolysis and regulation of neuron differentiation in AD,^5^ immune response and blood coagulation in DLB,^6^ and acute inflammatory response and response to axonal injury in FTD.^7^ Moreover, these studies identified proteins with differential expression in CSF compared to controls including microtubule‐associated protein tau (MAPT) and pyruvate kinase M1/M2 (PKM) for AD, neuronal pentraxin 2 (NPTX2) and neurosecretory protein VGF for DLB, and chitinase‐3‐like 1 protein (CHI3L1) for FTD.^5^, ^6^, ^7^

To what extent dysregulated CSF proteins and their associated biological processes overlap between these dementias is unclear. Many studies compare the disease of interest only to controls, or to a combined group of other dementias, or only include other dementias in the replication phase of the study. This may hamper the identification of biomarkers that are specific to each type of dementia.

Here, we utilized CSF proteomics to identify differentially expressed proteins and their associated biological processes in a cohort including AD, DLB, and FTD. In addition, we included mild cognitive impairment (MCI) with and without abnormal CSF amyloid‐beta biomarkers (MCI Aβ+, MCI Aβ‐), to study proteomic changes in a pre‐dementia stage. We confirmed our findings in two independent cohorts and in a systematic review of previous proteomics studies.

## 2 METHODS

### 2.1 Study participants

The discovery and the replication 1 cohort were selected by independent clinicians with an interval of 1 year and consisted of participants from the Amsterdam Dementia Cohort (ADC). Participants from the replication 2 cohort were enrolled by the Center for Memory disturbances at the University hospital of Perugia.

All ADC participants were extensively screened, including physical, neurological and neuropsychological examination, Mini‐Mental State Examination (MMSE), electroencephalogram, magnetic resonance imaging, and lumbar puncture. Diagnoses were made in a multidisciplinary consensus meeting in accordance with clinical criteria for MCI,^8^ AD,^9^ DLB^10^ and FTD.^11^, ^12^, ^13^ The control group consisted of participants with subjective cognitive decline (SCD) and was characterized by having normal clinical, cognitive and AD biomarker (CSF Aβ_1–42_, t‐tau, and p‐tau_181_) status. The MCI group was later subdivided into amyloid‐beta negative (MCI Aβ‐) or positive (MCI Aβ+) based on CSF Aβ_1–42_ levels, after which the MCI Aβ‐ group in the replication 1 cohort was excluded due to low sample size (*n* = 6).

Participants of the replication 2 cohort were diagnosed according to applicable criteria.^8^, ^14^ The control group consisted of patients with other neurological diseases. MCI was further subdivided based on CSF Aβ_1–42_ levels.

**RESEARCH IN CONTEXT**

1. **Systematic review**: Cerebrospinal fluid (CSF) proteomics studies have reported alterations in protein levels associated with neurodegenerative diseases like Alzheimer's disease (AD), dementia with Lewy bodies (DLB), or frontotemporal dementia (FTD) that may have diagnostic potential. Still, to what extent these alterations are shared or specific remains unclear as most studies did not directly compare these dementias.
2. **Interpretation**: We identified and validated glycolytic proteins that were specifically upregulated in AD dementia and amyloid‐beta positive MCI. These proteins were correlated with CSF amyloid‐beta and tau biomarker levels. A systematic review further confirmed the disease specificity of these AD markers. In contrast, the majority of the identified and validated DLB and FTD proteins showed similar directions of change across these dementias.
3. **Future directions**: The reproducibility of glycolytic proteins in this and other studies warrants further research into the relationship between glycolysis and AD pathology. Clinically feasible immunoassays need to be developed for these protein markers to assess their diagnostic utility.

### 2.2 CSF collection and biomarker measurements

The collection and storage of CSF was performed in accordance with international guidelines.^15^, ^16^ CSF was collected via lumbar puncture between L3/L4, L4/L5 or L5/S1 vertebra in polypropylene tubes and centrifuged within 2 h at 1800 g at room temperature for 10 min. After centrifugation, the samples were aliquoted in polypropylene tubes and stored at −80°C. Sample collection at the University of Perugia was performed similarly, except for centrifugation which was performed at 2000 g for 10 min at 4°C.

In the ADC, CSF levels of Aβ_1–42_, total tau (t‐tau) and phosphorylated tau_181_ (p‐tau_181_) were determined using immunoassays from Innotest (Fujirebio) or Elecsys (Roche). Aβ_1–42_ values determined using Innotest assays were corrected for upward drift over time.^17^ A positive biomarker status for Aβ_1–42_, t‐tau and p‐tau_181_ was defined using cutoffs of < 813, > 375 and > 52 for Innotest measurements, and < 1000, > 235 and > 19 for Elecsys measurements, respectively. In the replication 2 cohort, CSF biomarker levels were measured using Innogenetics immunoassays with cut‐offs for positive biomarker status of < 550 for Aβ_1–42_, > 300 for t‐tau and > 60 for p‐tau_181_.

### 2.3 CSF MS‐proteomics

All details regarding sample preparation and measurement can be found in the supplementary method 1. Briefly, samples from the discovery and replication 1 cohort were processed using in‐gel and in‐solution digestion, respectively. Then, both cohorts were measured using DIA‐MS and the resulting measurements were processed using a data‐dependent acquisition (DDA) ‐based spectral library of pooled CSF samples. The replication 2 cohort was processed using in‐solution digestion and measured with DDA‐MS. Batch effects between cohorts were assessed using principal component analysis, but no batch correction was performed (Figure S1).

### 2.4 Differential protein expression analysis

Data analyses were performed in R (v4.3.2). Data was normalized using median centering followed by log2 transformation. Next, the proteins levels were z‐transformed using the mean and standard deviation of the SCD or control group for each cohort separately. Differences in protein levels between diagnostic groups were calculated using linear regression models with diagnostic group as predictor and protein level as outcome including age and sex as covariates (emmeans, v1.8.8). This was done for all pairwise group comparisons with and without age and sex as covariates (Tables S1–3 and 4–6, respectively). Proteins were considered differentially expressed based on p‐value (*p* < 0.05) regardless of effect size. False discovery rate (FDR) adjusted *p*‐values (Tables S1–6) and power analyses (Tables S7–9) are available in the supplementary materials.

### 2.5 Biological enrichment analyses

Biological enrichment analyses were performed with clusterProfiler (v4.10.1) using the enrichGO function. To reduce redundancy, biological terms were clustered using hierarchical clustering with a minimum cluster size of 3 using aPEAR (v1.0.0). For each identified cluster of processes, the entry with the lowest adjusted *p*‐value and at least 3 mapped proteins was plotted. Protein interaction networks were generated using the STRING database (v12.0) and visualized using Cytoscape (v3.9.1). Protein interaction clusters were identified using ClusterONE, and gene ontology analysis was performed with BiNGO (October 2023). The disease associations were visualized using Omics Visualizer.

### 2.6 Correlation analysis

Validated proteins were correlated across the total discovery cohort with age, MMSE and CSF AD biomarkers using Pearson correlations (rcorr(), Hmisc v. 5.2–3). *P*‐values were not adjusted for multiple comparisons, as this was an exploratory analysis.

### 2.7 Systematic literature review

PubMed was searched for CSF proteomics research with search terms: “cerebrospinal fluid proteomics” & “Alzheimer's disease” or “dementia with Lewy bodies” or “frontotemporal dementia” or “mild cognitive impairment” (searched May 2024). We included studies that: were original data of human *ante mortem* CSF, had summary protein statistics available, measured > 100 proteins regardless of method. For AD and MCI literature, we additionally required to have CSF AD biomarker supported diagnosis and a sample size of *n* ≥ 20 per group. For the full list of included studies see supplementary method 2. Proteins were matched across studies using UniProt identifiers and considered significantly different from controls if their p value was < 0.05.

## 3 RESULTS

### 3.1 Cohort characteristics

To identify biomarkers for different dementia types, proteomics was performed in three CSF cohorts (*n* = 110, 112, 78) (Table S10). The groups in the discovery cohort were age‐matched and had similar group sizes (*n*∼20). The AD group had the lowest median MMSE (median = 21) followed by DLB (23), MCI Aβ‐ (26), FTD (27) and MCI Aβ+ (27). The majority of the DLB patients were male (95%) and 32% were CSF Aβ_1–42_ positive. A portion of the FTD group was t‐tau and p‐tau_181_ positive, 35% and 20%, respectively. In the replication 1 cohort, the SCD group was significantly younger (51.3 ± 9.6) than the other diagnostic groups (means ranged 63.6–69.3), and the MCI Aβ+ group was larger (*n* = 35). Similarly, the controls of the replication 2 cohort were also younger (62.1 ± 7.6) than the other diagnostic groups (means ranged 67.6–70.4).

### 3.2 Proteomics identifies disease specific protein alterations

**FIGURE 1:**
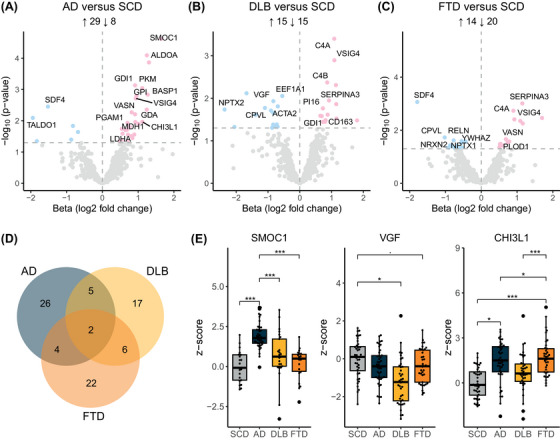
Discovery of differentially expressed proteins in AD, DLB, and FTD. (A–C) Volcano plots displaying the differentially regulated proteins between the different groups. Significantly (*p* < 0.05) increased and decreased proteins are shown in pink and blue, respectively. Selected proteins are labeled. (D) Venn diagram showing the number of unique and overlapping significantly dysregulated proteins in AD, DLB, and FTD compared to SCD. (E) Boxplots of three proteins with differential expression patterns across groups. Group levels were compared using linear regression (• = *p* < 0.1, * = *p* < 0.05, ** = *p* < 0.01, *** = *p* < 0.001). AD, Alzheimer's disease; DLB, dementia with Lewy bodies; FTD, frontotemporal dementia; SCD, subjective cognitive decline.

Next, proteins with different CSF levels between diagnostic groups were identified. For AD, 37 proteins exhibited altered levels (uncorrected *p* < 0.05, pFDR in Table S1–6) compared to SCD, of which 29 (78%) were upregulated (Figure 1A). The three most significantly altered proteins were involved in development and differentiation (SPARC related modular calcium‐binding [SMOC1]) (Figure 1E) and glycolysis (fructose‐bisphosphate aldolase A [ALDOA] and pyruvate kinase M [PKM]). Of the proteins dysregulated in AD, 26 (70%) were unaltered in DLB or FTD, and so possibly disease specific (Figure 1D). In the DLB group, 30 proteins displayed altered levels compared to SCD, of which 15 (50%) proteins were upregulated (Figure 1B). The three most significantly altered proteins were involved in innate immunity and complement pathways (Complement C4‐A [C4A], Complement C4‐A [C4B], and V‐set and immunoglobulin domain‐containing protein 4 [VSIG4]). Of the 30 dysregulated proteins, 17 (57%) were specific to DLB (Figure 1D). For FTD, 34 proteins were differentially expressed compared to SCD (Figure 1C), of which 14 (41%) were upregulated and 22 (65%) were disease specific (Figure 1D). The three most significantly altered proteins were involved in inflammation (Chitinase‐3‐like protein 1 [CHI3L1]) (Figure 1E), exocytosis (45 kDa calcium‐binding protein [SDF4]), and acute phase response (Alpha‐1‐antichymotrypsin [SERPINA3]). Two dysregulated proteins (C4A and VSIG4) were shared between AD, DLB, and FTD (Figure 1D) possibly reflecting shared biological processes.

### 3.3 Similar upregulation of immune responses occurs in AD, DLB, and FTD

**FIGURE 2:**
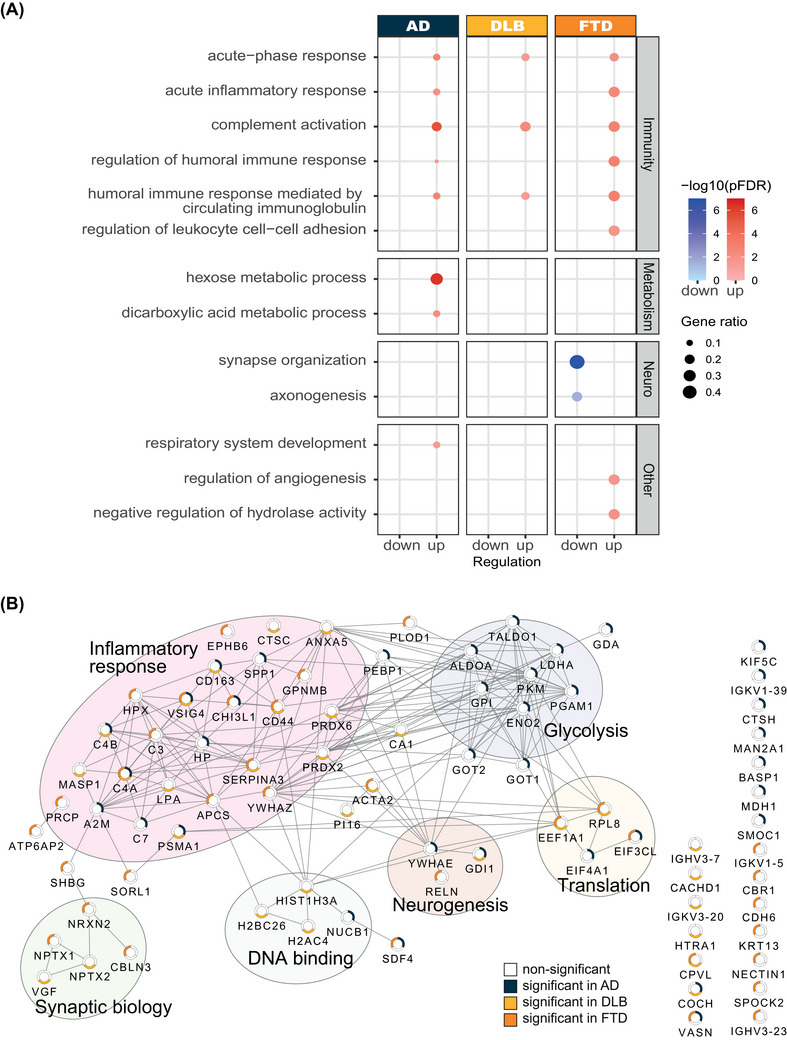
Biological mechanisms associated with AD, DLB, and FTD. (A) Biological process enrichment of the proteins upregulated (red) and downregulated (blue) in AD, DLB, and FTD compared to SCD. The size of the dots displays the gene ratio, and the color intensity shows the significance with lighter colors being less significant. The processes are grouped together into themes (Immunity, Metabolism, Neuro, and Other) to facilitate interpretation. (B) Interaction network of the identified dysregulated proteins. The colors in the pie‐chart denote for which groups the protein levels are significantly altered. Proteins without network association are shown in the lower right corner. AD, Alzheimer's disease; DLB, dementia with Lewy bodies; FTD, frontotemporal dementia; SCD, subjective cognitive decline.

Despite relatively minor overlap in proteins, AD, DLB, and FTD exhibited similar enrichment for immune related responses (Figure 2A). Shared upregulated processes included acute‐phase response, complement activation and humoral immune response related processes. Network analysis similarly identified a large inflammatory cluster (Figure 2B). While this cluster is shared across all three diseases, differences in disease association can be observed for the various proteins within the cluster. This may explain why, despite small overlap in differentially expressed proteins between these diseases, similar upregulation in immune processes is observed. Exclusively, FTD displayed a down‐regulation of synapse organization and axonogenesis and an upregulation of regulation of angiogenesis (Figure 2A). Furthermore, hexose metabolic processing was strongly and exclusively upregulated in AD.

### 3.4 Amyloid‐positive and amyloid‐negative MCI display dissimilar proteomic changes

**FIGURE 3:**
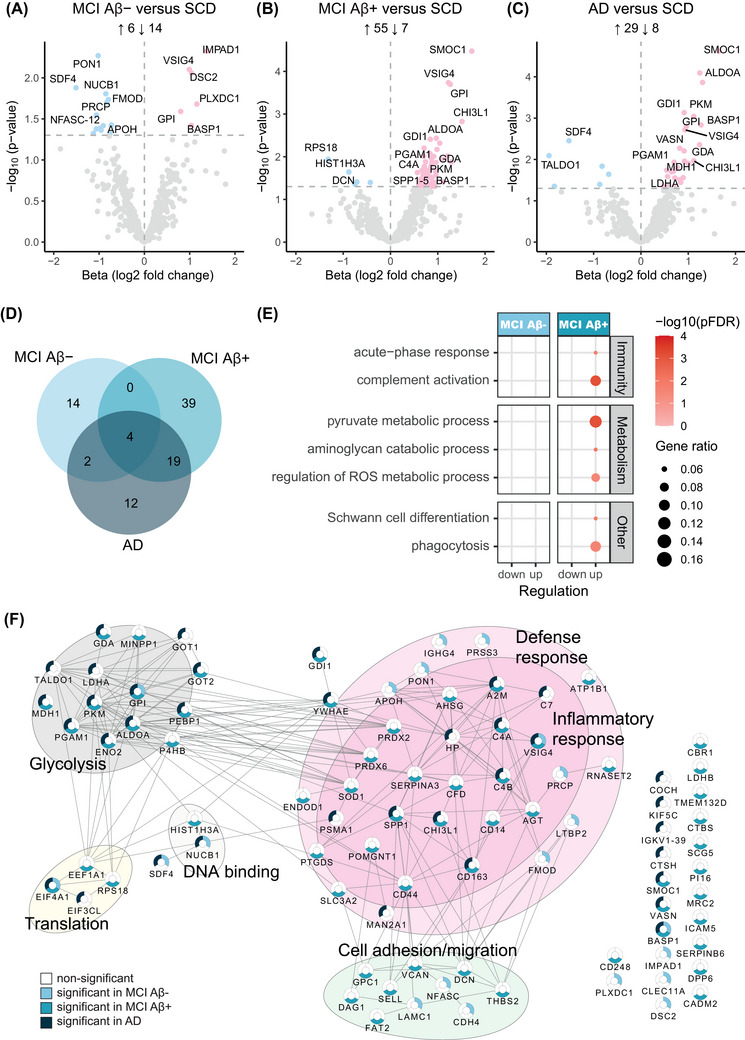
Differential protein expression in MCI Aβ‐ and MCI Aβ+. (A–C) Volcano plots depicting the differentially expressed proteins in MCI Aβ‐, MCI Aβ+, and AD compared to SCD. Significantly (*p* < 0.05) upregulated and downregulated proteins are pink and blue, respectively. Selected proteins are labeled. (D) Venn diagram showing the overlapping and unique dysregulated proteins for MCI Aβ‐, MCI Aβ+ and AD compared to SCD. (E) Biological process enrichment analysis of the dysregulated proteins shows only upregulated (red) processes in MCI Aβ+. (F) Interaction network of the identified dysregulated proteins for MCI Aβ‐, MCI Aβ+, and AD. The colors in the pie‐chart denote for which groups the protein levels are significantly altered. Proteins without network association are shown in the lower right corner. Aβ, amyloid‐beta; AD, Alzheimer's disease; MCI, mild cognitive impairment; SCD, subjective cognitive decline.

Next, protein expression changes in MCI Aβ+ and MCI Aβ‐ were assessed and compared. For MCI Aβ‐, 20 proteins exhibited altered levels compared to SCD (Figure 3A), of which the majority (14, 70%) were downregulated. MCI Aβ+ displayed more changes, with 62 dysregulated proteins, of which the majority (55, 89%) were upregulated compared to SCD (Figure 3B). The most significantly altered proteins in MCI Aβ+ were associated with differentiation (SMOC1), innate immunity (VSIG4), and glycolysis (glucose‐6‐phosphate isomerase [GPI]). MCI Aβ+ displayed a relatively large overlap of dysregulated proteins with AD (23, 37%) (Figure 3D), compared to DLB (12, 19%) and FTD (8, 13%). In contrast, only 4 (20%) differentially expressed proteins in MCI Aβ‐ overlapped with MCI Aβ+ (GPI, eukaryotic initiation factor 4A‐I [EIF4A1], VSIG4, brain acid soluble protein 1 [BASP1]). Moreover, 13 (65%) MCI Aβ‐ associated proteins did not overlap with those dysregulated in either AD, DLB, or FTD (Table S1).

MCI Aβ+ exhibited an upregulation of metabolic processes, like pyruvate metabolism and regulation of reactive oxygen species metabolism (Figure 3E). A glycolytic cluster was also identified in the network analysis (Figure 3F) and contained many shared AD (12/14) and MCI Aβ+ (10/14) associated proteins. This cluster displayed extensive connections with proteins of the inflammatory and defense response cluster, namely with peroxiredoxin‐2 (PRDX2), peroxiredoxin‐6 (PRDX6), and superoxide dismutase [Cu‐Zn] (SOD1), which were specifically associated with MCI Aβ+. While the defense and inflammatory response cluster contained a large portion (8/20) of the MCI Aβ‐ associated proteins it should be noted that these were not extensively connected. Reflecting this lack of connection, no biological processes were enriched in MCI Aβ‐.

### 3.5 Dysregulated proteins exhibit similar alterations across cohorts

**FIGURE 4:**
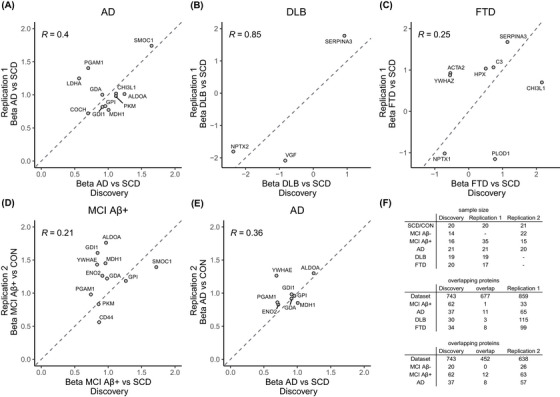
Replication of the differentially expressed proteins in the replication cohorts. (A–E) Scatter plots depicting the correlation of the beta coefficients (fold changes) between markers identified in the discovery and replication 1 and replication 2 cohorts. Only proteins significantly altered in both cohorts are displayed. ‘R’ denotes the Pearson correlation. Proteins that fall on the diagonal dashed line have an identical beta coefficient (fold change) in both cohorts. In (D), SERPINA3 is not shown as it falls outside the axis limits and displays opposite fold changes. (F) Table showing the sample sizes in each cohort (top) and the overlapping quantified proteins between the datasets and the overlapping altered proteins between cohorts (middle and bottom). SERPINA3, alpha‐1‐antichymotrypsin.

For AD, 11 dysregulated proteins were validated in the replication 1 cohort with concordant directions of change and a moderate correlation (rho = 0.40) of the fold changes observed in both cohorts (Figure 4A). Of these proteins, 7 (ALDOA, guanine deaminase [GDA], L‐lactate dehydrogenase A chain [LDHA], malate dehydrogenase cytoplasmic [MDH1], SMOC1, phosphoglycerate mutase 1 [PGAM1], and PKM) remained unaltered in DLB or FTD compared to SCD, confirming their specificity to AD. Furthermore, a combined panel of validated proteins could distinguish AD from DLB and FTD with similar accuracy compared to classical CSF AD biomarkers (ROC‐AUC = 0.90, DeLong's test *p* = 0.68, Figure S2). For DLB, three proteins, namely NPTX2, VGF, and SERPINA3, were validated (Figure 4B). NPTX2 displayed comparable effect sizes across cohorts, whereas VGF and SERPINA3 exhibited stronger effect sizes in the replication 1 cohort compared to the discovery cohort. For FTD, eight proteins were differentially expressed in the discovery and replication 1 cohort, of which five (NPTX1, CHI3L1, SERPINA3, Complement C3 [C3], and hemopexin [HPX]) displayed concordant directions of change across cohorts (Figure 4C).

For MCI Aβ+, one protein (GPI) was validated in the replication 1 cohort (Figure 4F), whereas 11 proteins were validated in the replication 2 cohort (Figure 4D). Of these 11 proteins, many, including ALDOA, GDA, MDH1, and PGAM1, overlapped with the validated AD proteins. For AD, 8 proteins replicated between the discovery and replication 2 cohort (Figure 4E), including ALDOA, GDA, GDI1, GPI, MDH1, and PGAM1 which have now been found across three cohorts. No MCI Aβ‐ proteins validated in the replication 2 cohort, despite the large overlap in identified proteins (Figure 4F).

### 3.6 Validated AD proteins correlate with CSF amyloid and tau markers

**FIGURE 5:**
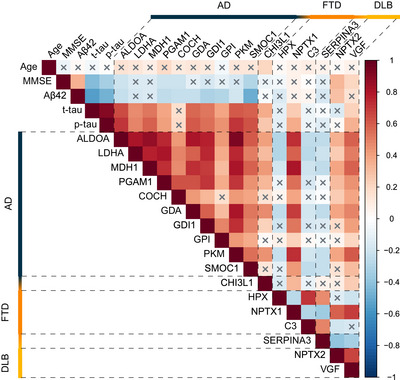
Correlation matrix of validated proteins with age, MMSE and AD biomarkers in the discovery cohort. The heatmap shows the Spearman's correlation of the validated proteins with age, MMSE and CSF Aβ1–42, t‐tau, and p‐tau181 in the discovery cohort without group stratification. Non‐significant correlations (*p* > 0.05) are denoted by a gray cross. Aβ, amyloid‐beta; AD, Alzheimer's disease; CSF, cerebrospinal fluid; MMSE, Mini‐Mental State Examination; p‐tau, phosphorylated tau; t‐tau, total tau.

Next, we performed a correlation analyses for the validated proteins with age, MMSE and AD biomarkers in the discovery cohort (Figure 5). The majority of the validated AD proteins exhibited correlations with CSF AD biomarkers. All proteins, except cochlin (COCH), displayed a positive correlation with CSF t‐tau and p‐tau_181_ levels and most, except LDHA, MDH1, and CHI3L1, exhibited a negative correlation with CSF Aβ_1–42_. ALDOA, PGAM1, COCH, and SMOC1 were negatively correlated with MMSE and CHI3L1 was positively correlated with age. For the validated DLB proteins, NPTX2 displayed a positive correlation with MMSE and CSF Aβ_1–42_ levels and VGF displayed a positive correlation with MMSE, CSF t‐tau, and p‐tau_181_ levels. The majority of the validated FTD proteins did not display significant correlations, except CHI3L1 and NPTX1, which were positively correlated with CSF Aβ_1–42_ levels.

### 3.7 Proteomics literature supports the dysregulated proteins in AD, DLB, and FTD

**FIGURE 6:**
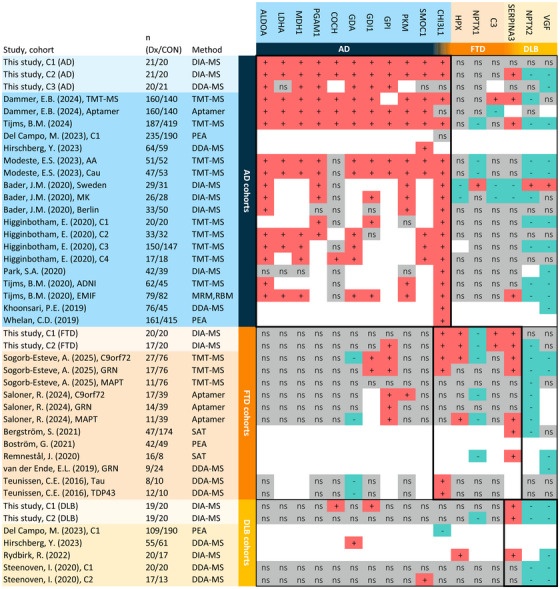
Replication of the identified AD, DLB, and FTD markers in proteomics literature. CSF proteomics literature was reviewed to assess the replication and disease specificity of the identified dysregulated proteins. Studies and cohorts are displayed on the vertical axis and proteins are displayed on the horizontal axis. Cells are colored according to their change compared to control (‘+’ = upregulated, ‘‐’ = downregulated, ‘ns’ = non‐significant (p ≥ 0.05), blank = not measured). If multiple cohorts from the same study are included, they are named or numbered similar to the original article. Sample sizes are given for disease (Dx) and control (CON) groups. Studies were included regardless of the type of proteomics method used. AD, Alzheimer's disease; aptamer, single stranded DNA aptamers; CSF, cerebrospinal fluid; DDA‐MS, data dependent acquisition mass spectrometry; DIA‐MS, data independent acquisition mass spectrometry; DLB, dementia with Lewy bodies; FTD, frontotemporal dementia; MRM, multiple reaction monitoring mass spectrometry; PEA, proximity extension assay; RBM, Rules‐Based Medicine; SAT, suspension array technology; TMT‐MS, tandem mass tags mass spectrometry.

Finally, a systematic review was performed for the validated proteins, which further confirm the upregulation for all validated AD proteins in four or more cohorts (Figure 6). In agreement with our findings, ALDOA, LDHA, MDH1, and PGAM1 were unaltered in DLB or FTD literature. In addition, ALDOA, MDH1, and PGAM1 were also upregulated in MCI Aβ+ in at least two previously published cohorts (Figure S3). For FTD, four out of five proteins have been identified in previous FTD literature with concordant directions of change as in our data. Notably, dysregulation of NPTX1 and SERPINA3 has now been observed using three different proteomics methods. HPX and C3, which were upregulated in FTD, were downregulated or displayed varying changes in AD literature, indicating that these markers may potentially be useful to distinguish FTD and AD. Lastly, literature confirmed the dysregulation of NPTX2, VGF, and SERPINA3 in DLB. However, similar changes in these proteins have been observed frequently in AD and FTD studies, indicating that these changes are not specific to DLB.

## 4 DISCUSSION

Fluid biomarkers could aid in the differential diagnosis of dementia. To this end we performed untargeted proteomics on CSF of three cohorts including patients with AD, DLB, FTD, MCI Aβ‐, and MCI Aβ+. Group specific protein changes were identified and validated for AD and FTD, whereas for DLB, only nonspecific changes validated across cohorts. In addition, an extensive systematic review including all disease groups and encompassing multiple proteomics methods was undertaken. This review further confirmed our findings and highlighted proteins that may have potential as a differential CSF biomarker.

We identified and validated 11 upregulated proteins for AD, of which many were associated with the glycolysis pathway and, similar to literature,^18^ were correlated with CSF Aβ_1–42_, t‐tau, and p‐tau_181_ levels. Previous analysis of brain tissue demonstrated an upregulation of glucose metabolic proteins in AD, possibly linked to astrocyte and microglial activation.^19^ A previous study reported that microglial activation caused by exposure to amyloid‐beta is associated with a metabolic shift favoring glycolysis.^20^ This may explain the observed glycolytic signature in AD CSF, but additional research is required to link findings in CSF to microglial activation. We and others observed that glycolytic proteins such as ALDOA, MDH1, and PGAM1 are upregulated in AD and MCI Aβ+ while remaining unaltered in other dementias,^21^, ^22^, ^23^, ^24^ suggesting that these proteins could serve as AD specific biomarkers across multiple disease stages.

Of note, most of the AD and MCI Aβ+ proteins were unaltered in MCI Aβ‐, providing further support that AD did not cause MCI in these individuals. Proteomic changes in the MCI Aβ‐ group validated poorly in the replication 2 cohort. This may be due to small sample sizes and differences in cohort selection resulting in age and sex differences. Additionally, the underlying cause of cognitive impairment in this group remains unclear, which may vary between individuals and not always be neurodegenerative. Future studies should aim to increase sample size in order to study potential underlying mechanisms in MCI Aβ‐.

In agreement with previous studies,^6^, ^25^, ^26^, ^27^ we found lower CSF levels of VGF and NPTX2 and higher SERPINA3 levels in DLB. We found VGF to be positively correlated with both MMSE and CSF t‐tau and p‐tau_181_ levels, which seems paradoxical as higher tau levels are usually related to worse MMSE scores. However, this has been observed before in AD and Lewy body disease.^28^, ^29^ Previous research suggests that the association between CSF VGF levels and longitudinal cognitive decline may be independent of p‐tau_181_.^30^ It has been hypothesized that VGF and NPTX2 levels increase as a protective mechanism in response to AD pathology, and that cognitive decline may ensue when this mechanism fails and these protein levels start to decline.^29^, ^30^ In line with this, VGF has been found increased in asymptomatic AD compared to controls, but decreased in symptomatic AD compared to controls.^31^ In comparison, less is known about SERPINA3, but it has been linked to AD, multiple system atrophy, and Parkinson's disease.^32^, ^33^, ^34^, ^35^, ^36^, ^37^ Early studies have shown an upregulation of SERPINA3 in CSF of AD and DLB correlating with blood brain barrier dysfunction.^38^ This could explain why SERPINA3 was not specific to DLB in our data, as blood–brain barrier dysfunction is a common finding in aging and neurodegenerative disease.^39^, ^40^

For FTD, we found altered levels of CHI3L1 (also called YKL‐40), HPX, NPTX1, C3, and SERPINA3. Notably, the FTD groups had different phenotypic distributions between cohorts, suggesting that these markers may be shared between different phenotypic variants of FTD. C3 and HPX may warrant further study as they were specifically upregulated in FTD. Literature supports the upregulation of HPX and C3 in FTD patients, as increased HPX levels have been found in brain^41^ and increased C3 levels have been found in CSF, plasma, and serum.^42^, ^43^, ^44^, ^45^ In DLB, CSF levels of HPX and C3 were not significantly altered compared to controls.^46^ In AD, reports on CSF levels of HPX and C3 have been mixed, with both reports on increased and unaltered levels of HPX,^47^, ^48^ and increased and decreased levels of C3 compared to controls.^5^, ^48^, ^49^, ^50^ Thus, while studies in DLB show no significant changes for C3 and HPX, studies in AD yield mixed results and additional research is necessary to determine the ability of these markers to detect FTD.

While AD diagnosis was supported by CSF biomarkers, DLB and FTD diagnosis was based solely on clinical assessment due to a lack of available disease specific biomarkers. Consequently, the biological heterogeneity may be higher and the diagnostic accuracy may be lower in DLB and FTD compared to AD, which may explain why more disease specific proteins were identified and validated for AD. Future studies should include biologically defined cohorts, using fluid biomarkers in AD, α‐synuclein seeding assays in DLB, and *post mortem* pathological examination or genetic testing in FTD. In addition, future studies should include larger sample sizes, as the moderate group sizes used in this study (*n*∼20) may have obscured subtle proteomic changes. Furthermore, future studies should include sex balanced cohorts, as the proteomic changes we observed in DLB may have been driven by a high predominance of male sex. Lastly, differences in sample processing and data acquisition may have complicated cross‐cohort comparisons. Still, we validated protein alterations in at least two independent cohorts, and found high concordance with existing literature indicating that the validated protein markers are robustly related to these diseases.

## 5 CONCLUSION

This study presents multiple unique multi‐disease cohorts which have been measured using reliable untargeted mass spectrometry‐based proteomics. This allowed us to compare changes in biological processes across diseases and disease stages and to study which protein level alterations were unique to AD, DLB, and FTD. We discovered that immune related processes are upregulated across these neurodegenerative diseases. We identified three disease specific glycolytic protein markers for AD (ALDOA, MDH1, PGAM1), which are altered in both the dementia and prodromal stage. In addition, we identified proteins (C3 and HPX) which may potentially be specifically altered in FTD.

## CONFLICT OF INTEREST STATEMENT

L.V. is supported by grant funding/collaborative study and consultancy/speaker fees from ZonMw (VENI grant), Amsterdam UMC (Startergrant) Stichting Dioraphte (biobank DemenTree), Olink, Lilly, and Roche; all paid to her institution. D.C. received travel grants from Fujirebio. L.P. served as Member of Advisory Boards for Fujirebio, IBL, Roche, and Merck. A.W.L. participated in advisory boards from Almirall, Biogen, Eisai, Fujirebio‐Europe, Grifols, Novartis, Roche, Otsuka Pharmaceutical, Nutricia, Zambón, y NovoNordisk. Y.A.L.P. receives funding from the Dioraphte Foundation, Health Holland, and ZonMW. C.E.T. has research contracts with Acumen, ADx Neurosciences, AC‐Immune, Alamar, Aribio, Axon Neurosciences, Beckman–Coulter, BioConnect, Bioorchestra, Brainstorm Therapeutics, C2N diagnostics, Celgene, Cognition Therapeutics, EIP Pharma, Eisai, Eli Lilly, Fujirebio, Instant Nano Biosensors, Merck, Muna, Novo Nordisk, Olink, PeopleBio, Quanterix, Roche, Toyama, Vaccinex, Vivoryon. C.E.T. is editor in chief of Alzheimer Research and Therapy, and serves on editorial boards of Molecular Neurodegeneration, Alzheimer's & Dementia, Neurology: Neuroimmunology & Neuroinflammation, Medidact Neurologie/Springer, and is committee member to define guidelines for Cognitive disturbances, and one for acute Neurology in the Netherlands. C.E.T. has consultancy/speaker contracts for Aribio, Biogen, Beckman–Coulter, Cognition Therapeutics, Danaher, Eisai, Eli Lilly, Janssen, Merck, Novo Nordisk, Novartis, Olink, Roche, Sanofi and Veravas. All other authors declare no competing interests.

## CONSENT STATEMENT

All study participants or their representative provided written informed consent for the scientific use of their data and biofluids.

## Supporting information



Supporting Information

Supporting Information

Supporting Information
