# Supplementary material for: Discovery of cerebrospinal fluid biomarkers for different dementias using mass spectrometry‐based proteomics
Source: Alzheimers Dement (Amst). 2026 Apr 12;18(2):e70278. doi: 10.1002/dad2.70278 (PMC13071172; doi:10.1002/dad2.70278)
Supplement: Supplementary file 2 — Supporting Information [file DAD2-18-e70278-s002.docx]

Discovery of cerebrospinal fluid biomarkers for different dementias using mass spectrometry-based proteomics

Marijke E. Stokkel, Lisa Vermunt, Jaco C. Knol, Davide Chiasserini, Lucilla Parnetti, Sander R. Piersma, Thang V. Pham, Richard R. de Goeij-de Haas, Afina W. Lemstra, Yolande A.L. Pijnenburg, Pieter J. Visser, Betty M. Tijms, Charlotte E. Teunissen, Connie R. Jimenez

Contents supplementary materials

[Supplementary method 1: Mass spectrometry proteomics 2](#_Toc214447413)

[1.1. CSF sample processing 2](#_Toc214447414)

[1.2. DIA-MS spectral library preparation 3](#_Toc214447415)

[1.3. Cohort nanoLC-MS/MS measurements 4](#_Toc214447416)

[1.4. Data processing, protein identification & quantification 5](#_Toc214447417)

[Supplementary method 2: Systematic review 6](#_Toc214447418)

[Supplementary table 10. Cohort characteristics 8](#_Toc214447419)

[Supplementary figure 2. ROC curves 11](#_Toc214447420)

[Supplementary figure 3. Replication of MCI markers in proteomics literature 12](#_Toc214447421)

# Supplementary method 1: Mass spectrometry proteomics

- 1. CSF sample processing

DIA-MS library pools

The discovery and replication 1 cohort were analyzed using DIA-MS, for which a spectral library was generated using pooled CSF. Two CSF pools were made per diagnostic group, each consisting of 5 samples, making a total of 12 pools encompassing 60 samples. CSF pools were run on a NuPAGE 4-12% Bis-Tris gel (Invitrogen) and stained using Coomassie Brilliant Blue G-250 (Pierce). Gel processing was performed in a flow cabinet under keratin free conditions. The gel was incubated for 10 min in ABC solution (50 mM ammonium bicarbonate) followed by incubating twice in ABC/ACN solution (25 mM ammonium bicarbonate, 50% acetonitrile) for 10 min. Cysteines were reduced with DTT (10 mM 1,4-dithiothreitol, 50 mM ammonium bicarbonate) for 1 h at 56 °C. Next, the cysteines were alkylated for 45 min at room temperature in the dark with IAA (54 mM iodoacetamide, 50 mM ammonium bicarbonate). The gels were then washed for 10 min in ABC solution followed by incubation for 10 min in ABC/ACN, and lastly in ABC. Each gel lane was cut into 10 slices, and each slice was cut into 1 mm^3^ pieces and transferred to a microcentrifuge tube. The gel pieces were dehydrated by incubation with 300 µL ABC/ACN for 10 min at 400 rpm followed by centrifugal evaporation at 60 °C. Trypsin solution (6.3 ng/mL sequencing grade modified trypsin (Promega), 50 mM ammonium bicarbonate) was added to the dried gel pieces and incubated overnight at 37 °C. The peptides were isolated using three formic acid (FA) extractions, once using 1% FA and subsequently twice using FA/ACN (5% FA, 50% ACN). The peptides were desalted using a 10 mg OASIS HLB column (Waters) as described previously (17).

#### Discovery cohort

The samples of the discovery cohort were processed using in-gel digestion. 35 μL of CSF was mixed with 5 μL 4x LDS sample buffer (NuPAGE, Invitrogen), boiled and separated briefly on a 4-12% Bis-Tris NuPAGE Gel (Invitrogen), collecting all proteins in one gel band. The gels were then processed as described above, with one slice made per sample lane.

#### Replication 1 cohort

The samples of the replication 1 cohort were digested using in-solution digestion (5). 40 μL CSF was reduced and alkylated for 10 min at 95 ^o^C using 40 μL lysis buffer (1% w/v sodium-deoxycholate (SDC), 10 mM tris(2-carboxyethyl)phosphine (TCEP), 40 mM chloroacetamide (CAA), 100 mM 4-(2-hydroxyethyl)-1-piperazineethanesulfonic acid (HEPES), pH 8.0). The samples were cooled prior to the addition of trypsin solution (1:10 w/w in 50 μL ABC) and incubated overnight at 37 ^o^C in a thermomixer. Detergents were removed from the samples using ethyl acetate (EtOAc) extraction. After addition of 40.6 μL 20% TFA, the samples were vortexed and left on ice for 5 min to precipitate. The precipitate was dissolved by addition of 500 μL EtOAc, after which the samples were vortexed and centrifuged (30 s, 10000 rcf) and the upper layer of EtOAc was removed. This step was performed twice. The water layer (700 μL) was transferred to a new low protein binding tube and 200 μL 0.1% TFA was added. The samples were dried in a vacuum centrifuge and desalted as described above.

#### Replication 2 cohort

The replication 2 cohort was processed using high abundant protein depletion and in-solution digestion. High abundant proteins were depleted by filtering 0.5 mL CSF using MARS spin filters (Agilent), followed by concentration using 3kDa filters (Millipore, Billerica, CA, USA). The concentrated CSF samples (20 μL) were reduced by incubation with 40 μL 9M Urea, 1M TRIS-HCl and 6 μL 200 mM DTT for 30 minutes at 56 ^o^C. Alkylation was performed at a final concentration of 45 mM IAA for 30 minutes at room temperature in the dark. DTT was added once more (final concentration 20 mM) and incubated for 30 minutes at room temperature. LysC was added (1:50 w/w) and left to incubate for 4 h at 37 ^o^C, followed by incubation with trypsin (1:50 w/w) overnight at 37 ^o^C. After digestion the peptide samples were acidified using 20 μL of 10% formic acid and purified using C18 chromatography. Desalted peptides were dried in a vacuum centrifuge.

- 1. DIA-MS spectral library preparation

#### Data-dependent acquisition LC-MS/MS

Tryptic peptides were separated using an Ultimate 3000 nanoLC-MS/MS system (Thermo Fisher Scientific) equipped with a 50 cm × 75 μm ID Acclaim Pepmap (C18, 1.9 μm) column. After injection, peptides were trapped at 3 μL/min on a 10 mm × 75 μm ID Acclaim Pepmap trap at 2% buffer B (buffer A: 0.1% FA, buffer B: 80% ACN, 0.1% FA). The peptides were separated at 300 nL/min in a 10–40% buffer B gradient in 110 min (140 min inject-to-inject) at 40°C. Eluting peptides were ionized at a potential of +2 kVa into a Q Exactive HF mass spectrometer (Thermo Fisher Scientific).

Intact masses were measured from m/z 350-1400 at resolution 120,000 (at m/z 200) in the Orbitrap using an AGC target value of 3E6 charges and a maxIT of 100 ms. The top 15 peptide signals (charge states 2+ and higher) were submitted to MS/MS in the higher-energy collision (HCD) cell (1.4 amu isolation width, 26% normalized collision energy). MS/MS spectra were acquired at resolution 15,000 (at m/z 200) in the Orbitrap using an AGC target value of 1E6 charges, a maxIT of 64 ms, and an underfill ratio of 0.1%. Dynamic exclusion was applied with a repeat count of 1 and an exclusion time of 30 s.

#### Data processing

The MS/MS spectra acquired in data-dependent acquisition (DDA) mode were analyzed using MaxQuant version 1.6.10.43 and searched against the Swissprot human reference proteome (cannonical_and_isoform.FASTA, downloaded January 2021). Enzyme specificity was set to trypsin, allowing up to two missed cleavages and a minimum peptide length of 7 amino acids. Cysteine carbamidomethylation was set as fixed modification and methionine oxidation and N-terminal acetylation as variable modifications. Peptide precursor ions were searched with a maximum mass deviation of 4.5 ppm and fragment ions with a maximum mass deviation of 20 ppm. Peptide and protein identifications were filtered at an FDR of 1% using the decoy database strategy. The minimum Andromeda score for modified peptides was 40 and the corresponding minimum delta score was 6. The MaxQuant MSMS.txt file was used to generate a spectral library for DIA data analysis.

- 1. Cohort nanoLC-MS/MS measurements

#### Discovery and replication 1 cohort

The discovery and replication 1 cohort were measured using DIA-MS on Q Exactive HF mass spectrometer (Thermo Fisher Scientific). 1 µg total peptides was separated using the LC-MS setup described above. The DIA-MS method consisted of an MS1 scan from 350 to 1400 m/z at 120,000 resolution (AGC target of 3E6 and 60 ms injection time). For MS2, 24 variable size DIA segments were acquired at 30,000 resolution (AGC target 3E6 and auto for injection time). The DIA-MS method starting at 350 m/z included one window of 35 m/z, 20 windows of 25 m/z, 2 windows of 60 m/z and one window of 418 m/z, which ended at 1400 m/z. Normalized collision energy was set at 28. The spectra were recorded in centroid mode with a default charge state for MS2 set to 3+ and a first mass of 200 m/z. Spectral library DDA data files were acquired with the same LC settings as the DIA experiments.

Replication 2 cohort

The replication 2 cohort was measured using DDA-MS in a Q Exactive mass spectrometer (Thermo Scientific). Intact masses were measured at a resolution of 70,000 (at m/z 200) in the orbitrap using an AGC target value of 3E6 charges. The top 10 peptide ions (charge states 2+ and higher) were submitted to MS/MS in the HCD cell (4 amu isolation width, 25% normalized collision energy). MS/MS spectra were acquired at a resolution of 17,500 (at m/z 200) in the Orbitrap using an AGC target value of 2E5 charges and an underfill ratio of 0.1%. Dynamic exclusion was applied with a repeat count of 1 and an exclusion time of 30 s.

- 1. Data processing, protein identification & quantification

The DIA-MS data of the discovery and replication 1 cohort were searched in Spectronaut version 13.8 and 14.10, respectively (Biognosys, Schlieren, Switzerland) using default settings. In addition to a DDA-based spectral library, a second spectral library was generated from the DIA data using the direct DIA (dDIA) approach available in Spectronaut. For the final search of DIA data in Spectronaut, both libraries were assigned. Upon completion of the search, protein group level results were saved as Spectronaut report for further analysis. Q-value, review and decoy filters were applied during export. The search results were exported at the fragment ion level for MaxLFQ protein quantification using the R package iq. For the analysis of the DIA-MS cohorts, proteins were not filtered based on detection rate and missing values were not imputed.

The DDA-MS data of the replication 2 cohort was searched against the Uniprot human reference proteome using MaxQuant (version 1.4.1.2), similar to the data processing that was used for the generation of the DIA library. Proteins with a data presence lower than 50% were excluded from further analysis. Remaining missing values were not imputed.

# Supplementary method 2: Systematic review

AD references: (1-11)

DLB references: (4, 12-14)

FTD references: (15-21)

MCI references: (3, 11, 22-24)

1. Dammer EB, Shantaraman A, Ping L, Duong DM, Gerasimov ES, Ravindran SP, et al. Proteomic analysis of Alzheimer's disease cerebrospinal fluid reveals alterations associated with APOE epsilon4 and atomoxetine treatment. Sci Transl Med. 2024;16(753):eadn3504.

2. Tijms BM, Vromen EM, Mjaavatten O, Holstege H, Reus LM, van der Lee S, et al. Cerebrospinal fluid proteomics in patients with Alzheimer's disease reveals five molecular subtypes with distinct genetic risk profiles. Nat Aging. 2024;4(1):33-47.

3. Del Campo M, Peeters CFW, Johnson ECB, Vermunt L, Hok AHYS, van Nee M, et al. CSF proteome profiling across the Alzheimer's disease spectrum reflects the multifactorial nature of the disease and identifies specific biomarker panels. Nat Aging. 2022;2(11):1040-53.

4. Hirschberg Y, Valle-Tamayo N, Dols-Icardo O, Engelborghs S, Buelens B, Vandenbroucke RE, et al. Proteomic comparison between non-purified cerebrospinal fluid and cerebrospinal fluid-derived extracellular vesicles from patients with Alzheimer's, Parkinson's and Lewy body dementia. J Extracell Vesicles. 2023;12(12):e12383.

5. Modeste ES, Ping L, Watson CM, Duong DM, Dammer EB, Johnson ECB, et al. Quantitative proteomics of cerebrospinal fluid from African Americans and Caucasians reveals shared and divergent changes in Alzheimer's disease. Mol Neurodegener. 2023;18(1):48.

6. Bader JM, Geyer PE, Muller JB, Strauss MT, Koch M, Leypoldt F, et al. Proteome profiling in cerebrospinal fluid reveals novel biomarkers of Alzheimer's disease. Mol Syst Biol. 2020;16(6):e9356.

7. Higginbotham L, Ping L, Dammer EB, Duong DM, Zhou M, Gearing M, et al. Integrated proteomics reveals brain-based cerebrospinal fluid biomarkers in asymptomatic and symptomatic Alzheimer's disease. Sci Adv. 2020;6(43).

8. Park SA, Jung JM, Park JS, Lee JH, Park B, Kim HJ, et al. SWATH-MS analysis of cerebrospinal fluid to generate a robust battery of biomarkers for Alzheimer's disease. Sci Rep. 2020;10(1):7423.

9. Tijms BM, Gobom J, Reus L, Jansen I, Hong S, Dobricic V, et al. Pathophysiological subtypes of Alzheimer's disease based on cerebrospinal fluid proteomics. Brain. 2020;143(12):3776-92.

10. Khoonsari PE, Shevchenko G, Herman S, Remnestal J, Giedraitis V, Brundin R, et al. Improved Differential Diagnosis of Alzheimer's Disease by Integrating ELISA and Mass Spectrometry-Based Cerebrospinal Fluid Biomarkers. J Alzheimers Dis. 2019;67(2):639-51.

11. Whelan CD, Mattsson N, Nagle MW, Vijayaraghavan S, Hyde C, Janelidze S, et al. Multiplex proteomics identifies novel CSF and plasma biomarkers of early Alzheimer's disease. Acta Neuropathol Commun. 2019;7(1):169.

12. Del Campo M, Vermunt L, Peeters CFW, Sieben A, Hok AHYS, Lleo A, et al. CSF proteome profiling reveals biomarkers to discriminate dementia with Lewy bodies from Alzheimer s disease. Nat Commun. 2023;14(1):5635.

13. Rydbirk R, Ostergaard O, Folke J, Hempel C, DellaValle B, Andresen TL, et al. Brain proteome profiling implicates the complement and coagulation cascade in multiple system atrophy brain pathology. Cell Mol Life Sci. 2022;79(6):336.

14. van Steenoven I, Koel-Simmelink MJA, Vergouw LJM, Tijms BM, Piersma SR, Pham TV, et al. Identification of novel cerebrospinal fluid biomarker candidates for dementia with Lewy bodies: a proteomic approach. Mol Neurodegener. 2020;15(1):36.

15. Sogorb-Esteve A, Weiner S, Simren J, Swift IJ, Bocchetta M, Todd EG, et al. Proteomic analysis reveals distinct cerebrospinal fluid signatures across genetic frontotemporal dementia subtypes. Sci Transl Med. 2025;17(784):eadm9654.

16. Saloner R, Staffaroni A, Dammer E, Johnson ECB, Paolillo E, Wise A, et al. Large-scale network analysis of the cerebrospinal fluid proteome identifies molecular signatures of frontotemporal lobar degeneration. Res Sq. 2024.

17. Bergstrom S, Oijerstedt L, Remnestal J, Olofsson J, Ullgren A, Seelaar H, et al. A panel of CSF proteins separates genetic frontotemporal dementia from presymptomatic mutation carriers: a GENFI study. Mol Neurodegener. 2021;16(1):79.

18. Bostrom G, Freyhult E, Virhammar J, Alcolea D, Tumani H, Otto M, et al. Different Inflammatory Signatures in Alzheimer's Disease and Frontotemporal Dementia Cerebrospinal Fluid. J Alzheimers Dis. 2021;81(2):629-40.

19. Remnestal J, Oijerstedt L, Ullgren A, Olofsson J, Bergstrom S, Kultima K, et al. Altered levels of CSF proteins in patients with FTD, presymptomatic mutation carriers and non-carriers. Transl Neurodegener. 2020;9(1):27.

20. van der Ende EL, Meeter LH, Stingl C, van Rooij JGJ, Stoop MP, Nijholt DAT, et al. Novel CSF biomarkers in genetic frontotemporal dementia identified by proteomics. Ann Clin Transl Neurol. 2019;6(4):698-707.

21. Teunissen CE, Elias N, Koel-Simmelink MJ, Durieux-Lu S, Malekzadeh A, Pham TV, et al. Novel diagnostic cerebrospinal fluid biomarkers for pathologic subtypes of frontotemporal dementia identified by proteomics. Alzheimers Dement (Amst). 2016;2:86-94.

22. Delvenne A, Gobom J, Tijms B, Bos I, Reus LM, Dobricic V, et al. Cerebrospinal fluid proteomic profiling of individuals with mild cognitive impairment and suspected non-Alzheimer's disease pathophysiology. Alzheimers Dement. 2023;19(3):807-20.

23. Kamalian A, Ho SG, Patel M, Lewis A, Bakker A, Albert M, et al. Exploratory Assessment of Proteomic Network Changes in Cerebrospinal Fluid of Mild Cognitive Impairment Patients: A Pilot Study. Biomolecules. 2023;13(7).

24. Visser PJ, Reus LM, Gobom J, Jansen I, Dicks E, van der Lee SJ, et al. Correction: Cerebrospinal fluid tau levels are associated with abnormal neuronal plasticity markers in Alzheimer's disease. Mol Neurodegener. 2022;17(1):37.

# Supplementary table 10. Cohort characteristics

|  |  | n | Age, mean (sd) | Male, n (%) | MMSE, median [IQR] | E4 carrier, n (%) | FTD subgroup, n (%) | Aβ1-42+, n (%) | t-tau+, n (%) | p-tau181+, n (%) |
| --- | --- | --- | --- | --- | --- | --- | --- | --- | --- | --- |
| Discovery | SCD | 20 | 59.2 (3.5) | 10 (50.0) | 29.0 [27.5, 30.0]b,c,d,e,f | 7 (35.0) | n/a | 0 (0.0)c,d | 0 (0.0)c,d | 0 (0.0)c,d,e |
|  | MCI Aβ- | 14 | 57.2 (0.9)e | 4 (28.6)e | 25.5 [23.5, 27.5]a,d | 8 (57.1) | n/a | 0 (0.0)c,d | 3 (21.4)d | 4 (28.6)d |
|  | MCI Aβ+ | 16 | 58.6 (1.3)e | 10 (62.5) | 26.5 [24.8, 28.0]a,d | 12 (75.0)f | n/a | 16 (100.0)a,b,e,f | 11 (68.8)a | 12 (75.0)a,f |
|  | AD | 21 | 59.3 (3.1) | 12 (57.1) | 21.0 [16.0, 23.0]a,b,c,f | 16 (76.2)f | n/a | 21 (100.0)a,b,e,f | 21 (100.0)a,b,e,f | 21 (100.0)a,b,e,f |
|  | DLB | 19 | 61.4 (3.4)b,c | 18 (94.7)b | 23.0 [20.5, 26.0]a | 12 (63.2)f | n/a | 6 (31.6)c,d | 3 (16.7)d | 10 (52.6)a,d |
|  | FTD | 20 | 59.5 (7.7) | 10 (50.0) | 26.5 [24.8, 28.0]a,d | 2 (10.5)cde | bvFTD= 14 (100.0)  nfvPPA=0 (0.0)  svPPA= 0 (0.0)  PPA= 0 (0.0) | 1 (5.0)c,d | 7 (35.0)d | 4 (20.0)c,d |
| Replication 1 | SCD | 20 | 51.3 (9.6)c,d,e,f | 14 (70.0) | 28.5 [28.0, 29.0]c,d,e,f | 2 (10.0)c,e | n/a | 0 (0.0)c,d,e | 0 (0.0)c,d,e | 0 (0.0)c,d,e |
|  | MCI Aβ+ | 35 | 68.5 (6.0)a,f | 19 (54.3) | 26.0 [25.0, 28.0]a,d | 23 (65.7)a | n/a | 35 (100.0)a,e,f | 23 (65.7)a,d | 26 (74.3)a,e |
|  | AD | 21 | 66.3 (8.3)a | 10 (47.6) | 15.5 [13.8, 18.0]a,c,e,f | 8 (42.1) | n/a | 21 (100.0)a,e,f | 21 (100.0)a,c,e,f | 21 (100.0)a,e,f |
|  | DLB | 19 | 69.3 (8.0)a,f | 17 (89.5) | 23.0 [20.5, 26.0]a,c,d | 11 (57.9)a | n/a | 11 (57.9)a,c,d,f | 5 (26.3)d | 4 (21.1)c,d |
|  | FTD | 17 | 63.6 (5.9)a,c,e | 10 (58.8) | 24.5 [22.8, 26.2]a,d | 4 (26.7) | bvFTD= 3 (17.6)  nfvPPA=3 (17.6)  svPPA= 10 (58.8)  PPA= 2 (11.8) | 0 (0.0)c,d,e | 6 (35.3)d | 6 (35.3)d |
| Replication 2 | CN | 21 | 62.1 (7.6)b,d | 7 (33.3) | 29.0 [27.0, 29.0]b,c,d | NA | n/a | 0 (0.0)c,d | 3 (14.3)c,d | 1 (4.8)c,d |
|  | MCI Aβ- | 22 | 70.4 (6.3)a | 10 (45.5) | 24.5 [23.0, 27.0]a,d | NA | n/a | 0 (0.0)c,d | 7 (31.8)c,d | 6 (27.3)c,d |
|  | MCI Aβ+ | 15 | 67.6 (7.9) | 8 (53.3) | 25.0 [23.5, 27.0]a,d | NA | n/a | 15 (100.0)a,b | 15 (100.0)a,b | 15 (100.0)a,b |
|  | AD | 20 | 70.0 (8.2)a | 13 (65.0) | 17.0 [14.0, 20.0]a,b,c | NA | n/a | 18 (90.0)a,b | 20 (100.0)a,b | 20 (100.0)a,b |

Parametrically and non-parametrically distributed variables are denoted by their mean and standard deviation (SD) and their median and interquartile range (IQR), respectively. Pairwise group comparisons were performed within cohorts using Wilcoxon signed-rank test for non-parametric, t-test for parametric and Fisher test for categorical variables. Significant differences (Bonferroni adjusted p<0.05) between groups of the same cohort are highlighted. ‘NA’= not available, ‘n/a’=not applicable.

1. Differs from SCD/CN
2. Differs from MCI Aβ-
3. Differs from MCI Aβ+
4. Differs from AD
5. Differs from DLB
6. Differs from FTD


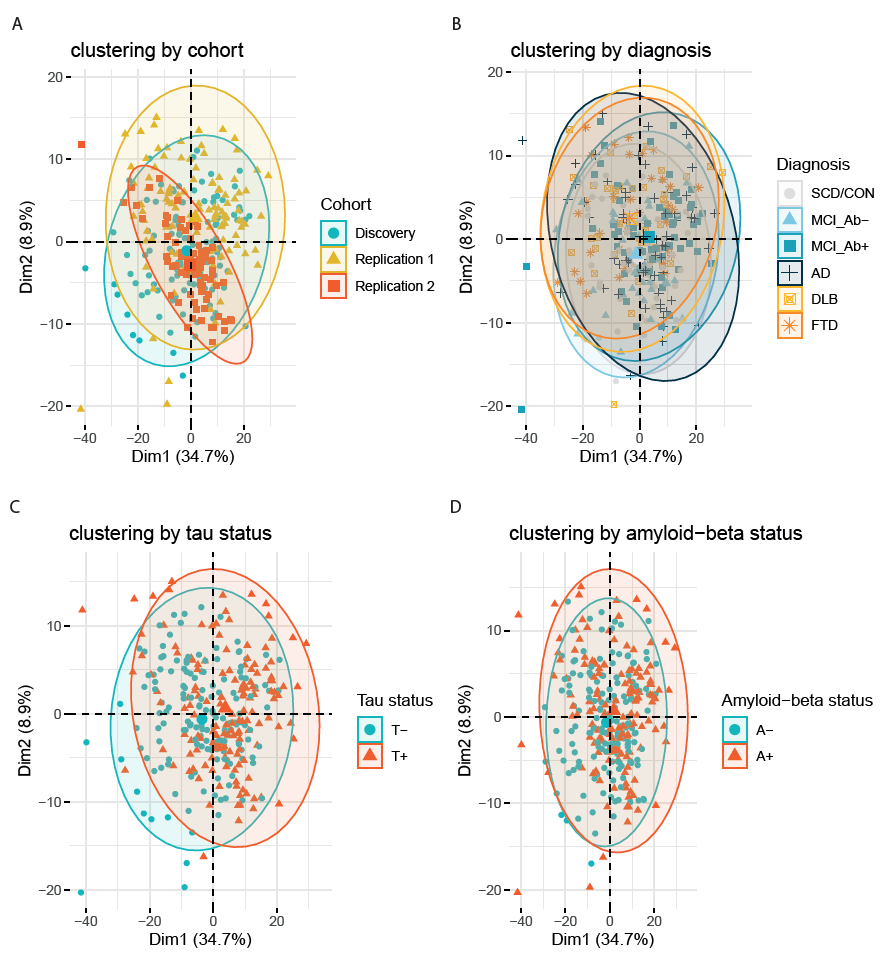
 **Supplementary figure 1. Principal component analysis**

Potential batch effects were assessed using principal component analysis (PCA). The analysis was performed on a combined dataset of all (3) cohorts, including all common proteins with fewer than 15% missing values (437 proteins). Missing protein values were imputed using missForest in R. The scatterplots are annotated for cohort (A), diagnostic group (B), CSF Tau status (C) and CSF Amyloid-beta_1-42_ status (D).


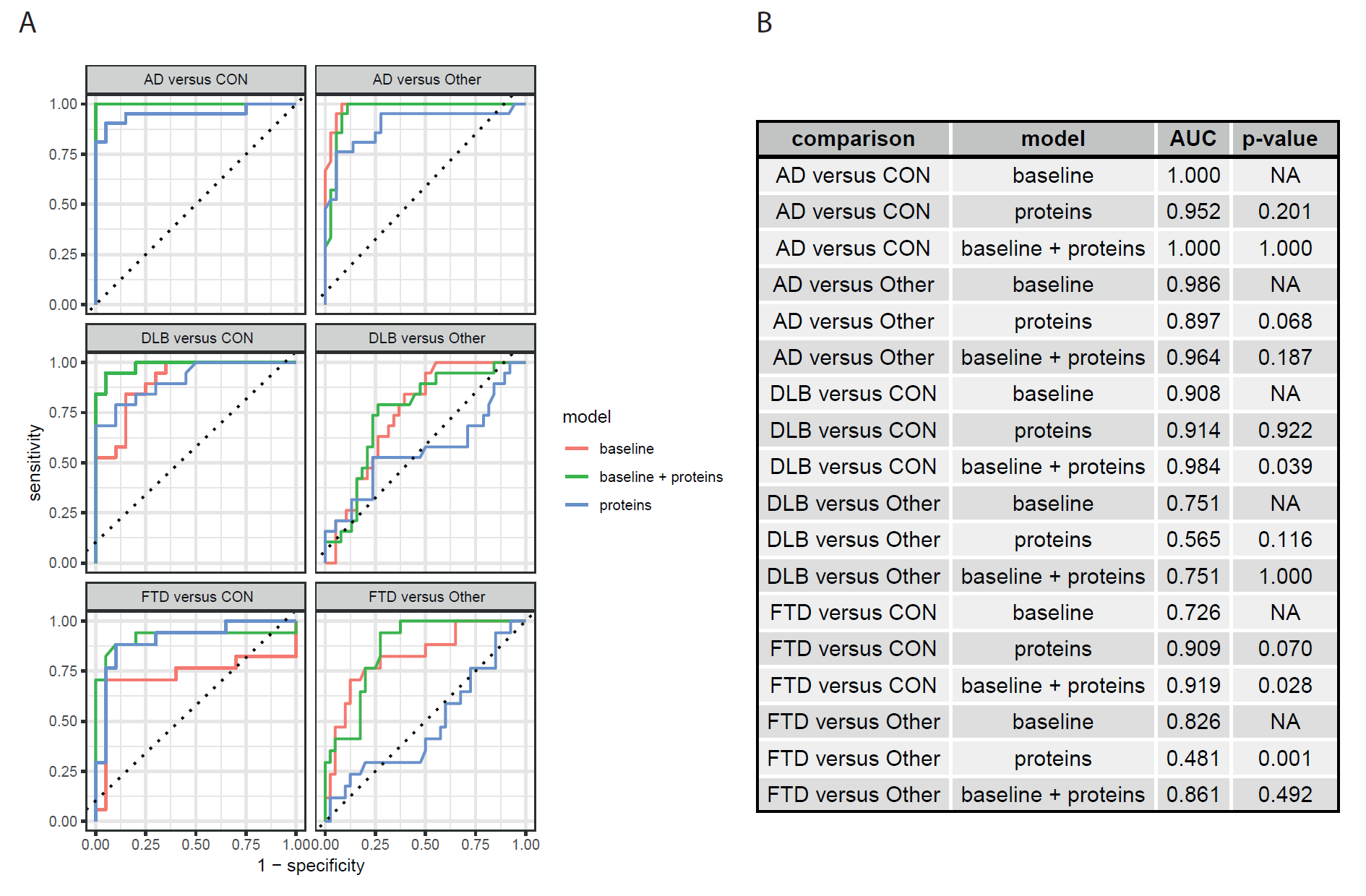


# Supplementary figure 2. ROC curves

Diagnostic accuracy of the validated proteins was tested using classification models. Random forests were trained using the discovery cohort and evaluated in the replication 1 cohort. Three models were compared, namely a baseline model using age, sex and CSF AD biomarkers, a protein model using the validated proteins for that group, and a combined model including baseline and protein variables. (A) ROC-curves depicting the diagnostic accuracy of the three models over 6 comparisons. (B) Table displaying the AUC of each model in each comparison. The AUCs of each model within the same comparison were compared to a baseline model using a DeLong’s test.


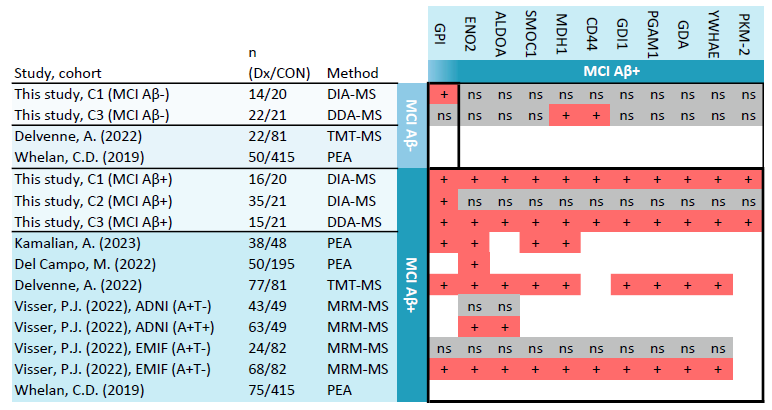


# Supplementary figure 3. Replication of MCI markers in proteomics literature

CSF proteomics literature was reviewed to assess the replication and specificity of the identified dysregulated proteins. Studies and cohorts are displayed on the vertical axis and proteins are displayed on the horizontal axis. Cells are colored according to their change compared to control (‘+’=upregulated, ‘-’=downregulated, ‘ns’=non-significant (p≥0.05), blank=not measured). If multiple cohorts from the same study are included, they are named or numbered similar to the original article. Sample sizes are given for disease (Dx) and control (CON) groups. Studies were included regardless of the type of proteomics method used. Abbreviations: DIA-MS=data independent acquisition mass spectrometry, DDA-MS= data dependent acquisition mass spectrometry, TMT-MS= tandem mass tags mass spectrometry, PEA= proximity extension assay, MRM= multiple reaction monitoring mass spectrometry.
